# Denture-Related Stomatitis Is Associated with Endothelial Dysfunction

**DOI:** 10.1155/2014/474016

**Published:** 2014-06-19

**Authors:** Joanna Maciąg, Grzegorz Osmenda, Daniel Nowakowski, Grzegorz Wilk, Anna Maciąg, Tomasz Mikołajczyk, Ryszard Nosalski, Agnieszka Sagan, Magdalena Filip, Mirosław Dróżdż, Jolanta Loster, Tomasz J. Guzik, Marta Cześnikiewicz-Guzik

**Affiliations:** ^1^Department of Prophylaxis and Experimental Dentistry, Institute of Dentistry, Jagiellonian University Medical College, Cracow, Poland; ^2^Department of Internal and Agricultural Medicine, Jagiellonian University Medical College, Cracow, Poland; ^3^Zbigniew Żak Voivodeship Dental Clinic, Cracow, Poland; ^4^Institute of Cardiovascular and Medical Sciences, University of Glasgow, UK; ^5^Department of Dental Prosthetics, Institute of Dentistry at Jagiellonian University Medical Collage, Cracow, Poland

## Abstract

Oral inflammation, such as periodontitis, can lead to endothelial dysfunction, accelerated atherosclerosis, and vascular dysfunction. The relationship between vascular dysfunction and other common forms of oral infections such as denture-related stomatitis (DRS) is unknown. Similar risk factors predispose to both conditions including smoking, diabetes, age, and obesity. Accordingly, we aimed to investigate endothelial function and major vascular disease risk factors in 44 consecutive patients with dentures with clinical and microbiological features of DRS (*n* = 20) and without DRS (*n* = 24). While there was a tendency for higher occurrence of diabetes and smoking, groups did not differ significantly in respect to major vascular disease risk factors. Groups did not differ in main ambulatory blood pressure, total cholesterol, or even CRP. Importantly, flow mediated dilatation (FMD) was significantly lower in DRS than in non-DRS subjects, while nitroglycerin induced vasorelaxation (NMD) or intima-media thickness (IMT) was similar. Interestingly, while triglyceride levels were normal in both groups, they were higher in DRS subjects, although they did not correlate with either FMD or NMD. *Conclusions*. Denture related stomatitis is associated with endothelial dysfunction in elderly patients with dentures. This is in part related to the fact that diabetes and smoking increase risk of both DRS and cardiovascular disease.

## 1. Introduction

Oral inflammation is an important element in the pathogenesis of vascular disease. In particular, large body of evidence has accumulated recently that chronic periodontitis is a potential novel risk factor for atherosclerosis and endothelial dysfunction [1–5]. Indeed, intensive treatment of chronic periodontitis alleviates endothelial dysfunction in a long-term follow-up, with clinical benefit lasting up to 6 months after intensive treatment [6]. The mechanisms of this association are not clearly defined but are most likely dependent on systemic inflammatory response, involving increased levels of IL-6, CRP, TNF-alpha, and other cytokines, which accompany periodontitis [6, 7]. Moreover, cellular immunity is also activated in periodontitis, including monocyte subpopulation shift as well as T cell activation with overproduction of interferon gamma and IL-17 [8].

While numerous studies have focused on the links between periodontitis and endothelial dysfunction, little is known about the links between other forms of oral infection and inflammation in the context of cardiovascular risk. In particular, denture-related stomatitis (DRS) is an inflammatory process of the oral mucosa in contact with a denture and is one of the most common diseases in elderly patients, affecting up to 70% of patients in the course of life [9, 10]. It is most common in complete prosthesis wearers, edentulous subjects [9–11]. The potential links are particularly worth addressing, as major risk factors for DRS include smoking, diabetes, age, and obesity, which coincide with risk of atherosclerosis and vascular disease [9, 12, 13]. Thus it is even more surprising that this problem of concomitant incidence of both conditions has not been studied up to date. Interestingly, the relationship of DRS to dyslipidemia is not known and female sex appears to predispose to higher occurrence [9]. Clinical symptoms of DRS include erythema and swelling of palatal mucosa, sometimes combined with subjective symptoms, such as dysgeusia or burning sensation. The aetiology of the DRS is multifactorial [9]. Long-term and continuous use of dentures and poor denture and oral hygiene habits promote the development of a biofilm, called denture plaque, on the surface of the prosthesis [9, 12].* Candida albicans* is fungal component of the physiological microflora of the human oral cavity [14, 15]; however, factors mentioned above may promote its excessive growth and, consequently, the development of infection and DRS.

While in periodontitis systemic activation of the immune system is very important in mediating increased cardiovascular risk, the extent of systemic response to DRS is poorly characterized. Systemic inflammation may affect vascular dysfunction in number of ways, which include activation of monocytes and T cells with overproduction of cytokines such as interferon *γ*, TNF-alpha, interleukin 6, or 17 [16], subsequently leading to atherosclerosis and hypertension [16–18] and increased cardiovascular risk. Interestingly, increased cardiovascular risk has been shown also for caries [19–21], as well as endodontic infection [22–26]. These diseases are all caused by bacterial infections, but, other microorganisms are also able to infect oral tissues. Relationship between fungal infection in oral cavity and systemic inflammatory response in context of vascular risk has not yet been studied. Therefore, the aim of this study was to determine whether the presence of DRS coincides with the clinical measures of vascular dysfunction, such as impaired endothelial function or elevated blood pressure.

## 2. Methods

### 2.1. Patients

Using 44 consecutive patients with dental prostheses for at least 6 months were included in this study. Their oral mucosa was examined by the dentist to clinically identify inflammation and DRS. The clinical signs of oral mucosa inflammation in DRS include erythema and swelling of palatal mucosa, sometimes combined with subjective symptoms, such as dysgeusia or burning sensation. These observations were confirmed by routine microbiological laboratory diagnostic tests for* Candida *species presence. Based on clinical and microbiological investigations, patients were divided into DRS (*n* = 20) group and non-DRS (*n* = 24) group. Diagnosis was confirmed by an independent observer. Control, non-DRS patients had clinically healthy oral mucosa and negative oral* Candida* swabs. Clinically healthy oral mucosa was a pale pink, smooth mucosal membrane without redness or swelling and with no pain or discomfort reported by patient. Exclusion criteria included acute inflammatory disorders other than DRS, neoplastic disease relapses or chemotherapy courses less than 5 years before the enrolment, and using antibiotics in less than 4 weeks or anti-inflammatory drugs (steroids and nonsteroidal, excluding aspirin in doses less than 80 mg) in less than 2 months before the enrolment. Patients with history of myocardial infarction, acute coronary incident or vascular inflammation in 5 weeks or less before the enrolment, chronic haematological disorders, and immunodeficiency or major medication changes during less than 5 weeks before or during study were also excluded. The study was approved by local ethics committee of Jagiellonian University. Informed consent was obtained from all patients and all work was conducted in accordance with the Declaration of Helsinki (1964).

### 2.2. Microbiological Investigations

Swabs were taken from the hard palate (between the second and third palatal fold). Samples were collected after an overnight fast and after at least 6 hours of continous denture usage, without the use of adhesives or rinsing the mouth with disinfectants. The material was collected in accordance with the general principles of microbial material collection.

### 2.3. Clinical Data

Patients' blood pressure (systolic, diastolic) was monitored for 24 hours using ambulatory blood pressure monitoring system (ABPM; SpaceLabs 90217, Ultralite device). Systolic diastolic and mean arterial pressures were recorded every 20 minutes for 24 hours. Day and night averages were calculated. One patient in control group did not agree to wear the ABPM monitor. Major risk factors for both atherosclerosis and DRS were recorded based on patient medical records and detailed patient history. Clinical risk factors were defined as follows: hyperlipidemia (total plasma cholesterol level > 5 mmol/L and/or triglycerides level > 1,7 mmol/L), diabetes (fasting glucose level ≥ 7 mmol/L or HbA1c > 6.5% or current treatment with insulin or oral hypoglycemic agents), hypertension (blood pressure ≥ 140/90 mmHg or current treatment with antihypertensive agents), and smoking (current or within last 6 months) based on [27]. Blood samples were obtained from antecubital vein and lipoprotein profile was assessed by routine diagnostic measurements of triglycerides, total cholesterol, low-(LDL), and high-(HDL) density lipoprotein cholesterol fractions. C-reactive protein (CRP) concentration was also assessed as in routine diagnostics.

### 2.4. Endothelial Function Measurement

Flow-mediated dilatation (FMD) method was used to determine the vascular endothelial function and NMD (nitroglycerine-mediated dilatation) for measuring endothelial-independent vasodilatation. Measurements were conducted using Toshiba Xario Diagnostic Ultrasound System after 1, 2, and 4-5 minutes after manometer cuff deflation or sublingual administration of nitroglycerine and presented as percentage of the diameter of the artery before intervention. Method validation in our laboratory has been described elsewhere [28]. Observers were blinded regarding oral status of the patients.

### 2.5. Subclinical Atherosclerosis Assessment

The measurements of intima-media thickness were performed in 12 different points (2 cm below common carotid arteries bulbs, ca. every 1 cm, omitting visible coronary plaques), on right and left common carotid artery, measuring the distance between the border between artery lumen and carotid artery intima and second bright line-m (border between media and adventitia) as described previously [28].

### 2.6. Statistical Analysis

Analysis was performed using Statsoft Statistica software. Compliance of the distribution of variables with normal distribution was tested by Shapiro-Wilk test. Most of the variables did not have normal distributions, and therefore the results are presented as medians and 25th (*Q*1), 75th (*Q*3) percentiles. For those variables nonparametric statistical tests were used, Mann-Whitney *U* test for continuous variables or, for dichotomous variables, *χ* ^2^ for the expected frequencies > 5 or *χ* ^2^ with Yates' correction for the expected frequencies < 5 with confirmation of Fisher's exact test and Spearman correlation. For variables with normal distribution Student's *t*-test was applied and data are presented as mean with standard deviation (SD). Method of presentation of results is given for each variable in the text. Values of *P* < 0, 05 were considered statistically significant.

## 3. Results

### 3.1. Clinical Risk Factors in Studied Groups

Both groups were balanced in terms of age, sex, body mass index (BMI) value, and antihypertensive treatment. There were more smokers and patients with diabetes mellitus (DM) in DRS group than in control group, although these differences were not statistically significant. Higher prevalence of DM and smoking in DRS group is consistent with epidemiologic data and is understandable, as both are recognized as a risk factor for developing DRS [13]. The proportion of males in both study groups was lower than expected for general population, which is consistent with the epidemiology of DRS, which is more common in females [9]. Patients characteristics are summarized in Table 1.

### 3.2. Blood Pressure in Denture Related Stomatitis

**Figure 1:**
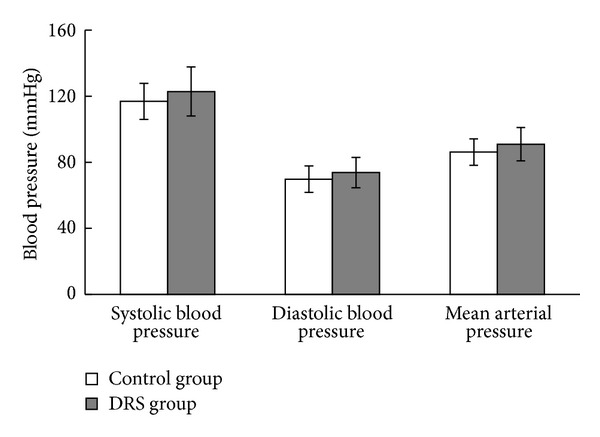
Ambulatory blood pressure parameters in control and DRS patients. Blood pressure parameters were assessed by 24 h measurement with ambulatory blood pressure monitoring system. Results are presented as mean (SD); *n* control group = 23, *n* DRS group = 20.

Ambulatory blood pressure monitoring has shown no significant differences in both mean systolic and mean diastolic blood pressure in DRS and control non-DRS group (Figure 1). Moreover, subsequent analysis of blood pressures during activity and rest periods did not show significant differences either (data not shown).

### 3.3. Vascular Function

**Figure 2:**
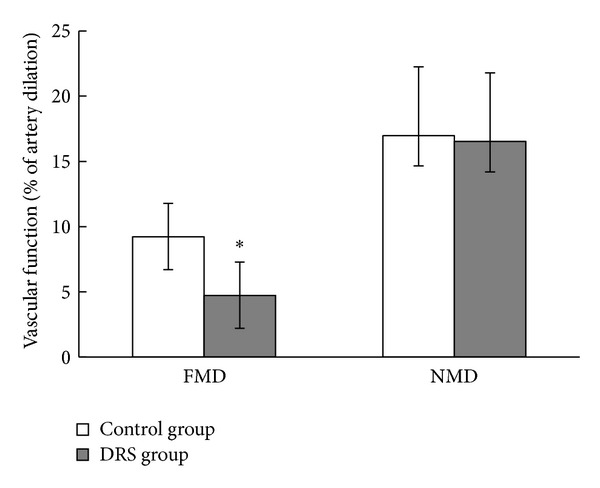
Vascular dysfunction in control and DRS. Vascular endothelium-dependent flow-mediated dilatation (FMD) and endothelium-independent nitroglycerin-mediated dilatation (NMD) parameters were assessed by ultrasonography. Results presented as median (*Q*1;* Q*2); ∗*P* < 0,005; *n* control group = 24, *n* DRS group = 20.

Flow-mediated dilatation measurements showed a significantly reduced median percentage of arterial dilation in response to flow in the DRS group in comparison with control patients (Figure 2). At the same time there was no difference between groups in endothelium-independent vasodilatation, NMD (Figure 2). There was no difference in baseline vessel diameter between control and DRS group (3,7 ± 0,8 mm versus 3,8 ± 0,7 mm; *P* = 0,4).

### 3.4. Subclinical and Clinical Atherosclerosis

**Figure 3:**
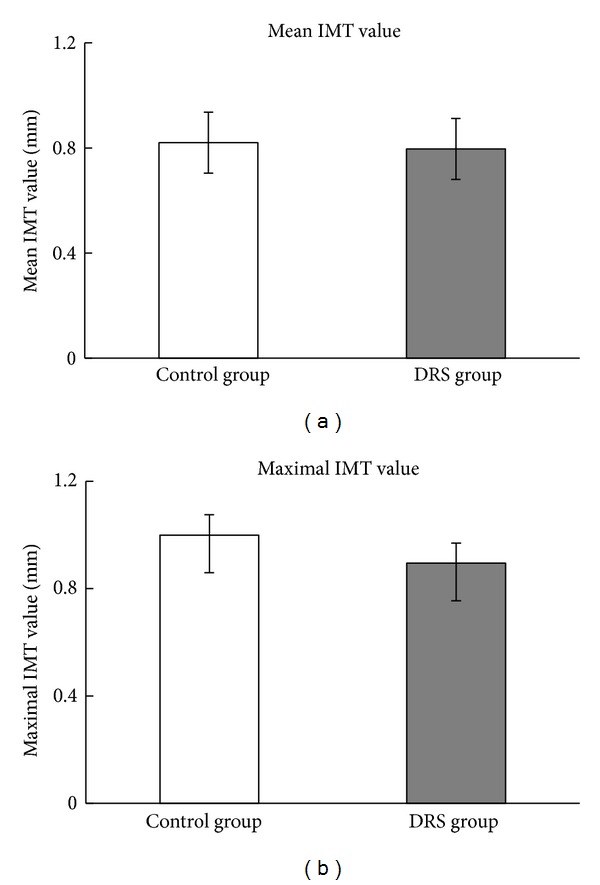
IMT measurements in DRS and control group. (a) Mean common carotid artery intima-media thickness. Results are presented as mean (SD); (b) maximal common carotid artery intima-media thickness. Results presented as median (*Q*1;* Q*2). (a) and (b): *n* control group = 24, *n* DRS group = 20.

Intima-media thickness evaluation showed no significant differences in either maximal or mean IMT in studied groups. It is important to point out that neither of the groups showed very high values of mean IMT (Figure 3). Moreover, presence of the atherosclerotic lesions of common carotid artery was equally distributed between groups; it was detected in 37.5% of patients from control group and in 35% of DRS group, *P* = 0, 86.

### 3.5. Plasma Lipid Profile and CRP

**Figure 4:**
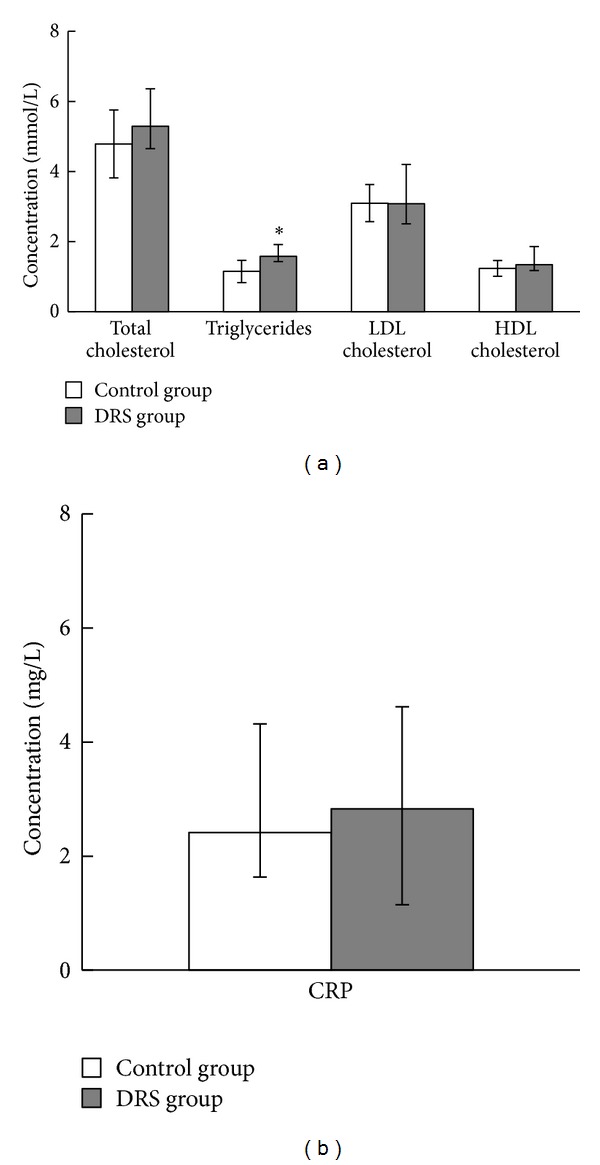
Plasma lipid profile and C-reactive protein levels in control and DRS patients. (a) Comparison of lipid profiles. Results are presented as median (*Q*1;* Q*2); ∗*P* < 0,05; (b) comparison of plasma CRP concentrations. Results are presented as median (*Q*1;* Q*2). (a) and (b): *n* control group = 24, *n* DRS group = 18.

**Figure 5:**
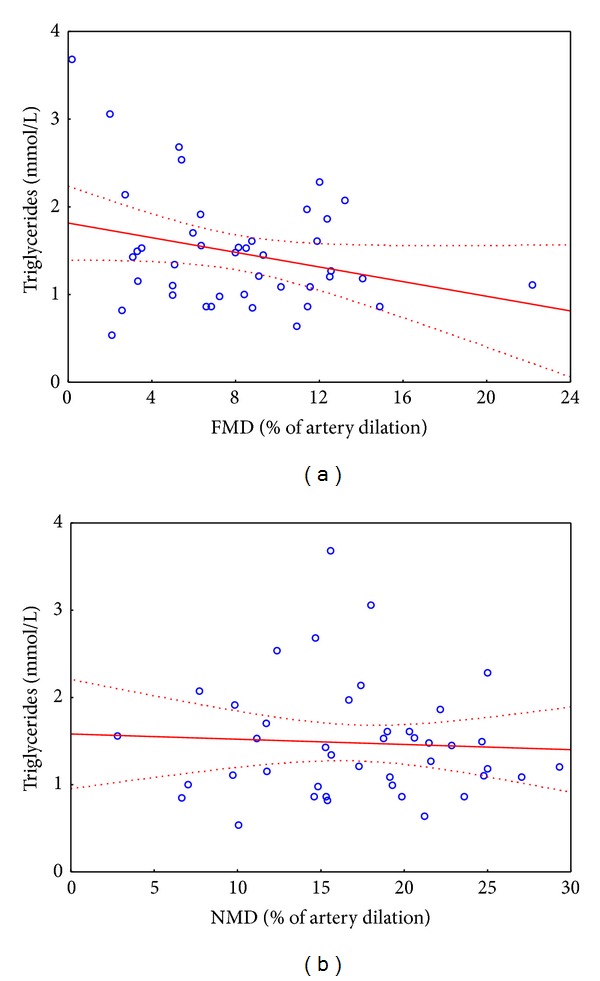
Spearman correlation between parameters of vascular function parameters and triglycerides levels. (a) Spearman correlation between FMD and triglycerides levels: *R* Spearman = −0,13, *P* = 0,42; (b) Spearman correlation between NMD and triglycerides levels: *R* Spearman = −0, 025, *P* = 0,87; (a) and (b): *n* = 42.

As the elevated blood triglycerides, LDL and total cholesterol levels and low HDL cholesterol levels are recognized as cardiovascular risk factors; we compared their concentrations in blood samples collected from patients with oral fungal infection and with healthy oral mucosa. We found that plasma levels of total, LDL, and HDL cholesterol were similar in both groups; however, triglycerides levels were significantly elevated in DRS group (Figure 4(a)). Surprisingly, CRP levels were similar between studied groups, indicating lack of significant component of systemic inflammation in DRS (Figure 4(b)). As the level of triglycerides was different between groups and this parameter may impact vascular function, we checked if there is a correlation between FMD or NMD and triglycerides levels. We found that these parameters were not correlated in case of FMD (*R* Spearman = −0,13, *P* = 0,42) and NMD (*R* Spearman = −0,025, *P* = 0,87) (Figure 5).

### 3.6. Subgroup Analysis in Female Subpopulation Only

As there was a much lower proportion of males in our study population, we performed an additional subgroup analysis in female population. It revealed that all studied vascular phenomena were observed to the same extent as in total studied population, including the difference in endothelium derived vasorelaxations (FMD (mean ± SD): 5,95 ± 3,80% in female DRS patients and 9,72 ± 3,31% in control subjects; *P* = 0,0032) and TG levels (median [Q1; Q2]: 1,61 [1,46; 1,99] in DRS versus 1,09 [0,86; 1,34] in non-DRS; *P* = 0,01).

## 4. Discussion

The oral health impact on the general health is evident. Oral infections and inflammation have been implicated in many disease entities, such as rheumatoid arthritis [29], obesity [30], negative pregnancy outcomes [31], DM [32], and even in epilepsy [33]. In particular, the role of oral inflammation and infection in the modulation of the risk of cardiovascular disorders has been well defined [1–5]. These studies have, however, focused mainly on periodontal inflammation and gingival bleeding. In the present study we investigated the relationships between denture-related stomatitis, a common oral inflammatory condition in elderly patients with endothelial dysfunction, blood pressure, and lipid profile. We observed that denture-related stomatitis which occurs in ca. 60% of patients wearing dentures, is associated with significant reduction of endothelial function, measured as nitric oxide bioavailability in a clinical flow-mediated dilatation study. Importantly, control, nitroglycerin induced endothelium-independent vasodilatation was not changed. As it is known that the severity of endothelial dysfunction correlates with the development of coronary artery disease and predicts future cardiovascular events [34], our results implicate that the presence of DRS may be associated with negative cardiovascular outcomes. Thus, such patients should be particularly carefully monitored in relation to their cardiovascular risk. Considering that DRS is one of the most common oral disorders in the elderly, occurring in 40–70% of subjects wearing dentures [9, 10], our finding may have important implications for clinical care of denture wearing patients. It is important to note, however, that majority of patients studied here were females which is consistent with the epidemiology of DRS, that is, more common in females [9]. We have also performed an additional analysis in female subpopulation only, which confirmed all major observations of this study.

While numerous previous studies have shown increased cardiovascular risk in subjects with oral inflammatory conditions such as periodontitis [1–5], endodontic infections [22–26], and even caries [19–21], this is the first study focusing on vascular dysfunction in elderly population of patients wearing dentures. This is important, while previous studies focused on bacteria-mediated, resulting from disturbances of physiological oral microflora diseases, we have primarily studied fungal infection, as DRS is most commonly associated with* Candida* infection.

Previous studies focused on a positive association between periodontitis and vascular endothelial dysfunction. Amar et al. and Blum et al. observed that subjects with advanced periodontal disease exhibit worse endothelial function when compared to the healthy controls [35, 36]. Blum et al. [36], along with others [6, 37], reported also an improvement of endothelial function as a long-term outcome of periodontal treatment. Tonetti et al. [6] in a landmark study has shown in a proper randomized controlled trial that such improvement provides clinical benefit for up to 6 months after intensive treatment.

The mechanisms of increased cardiovascular risk in oral inflammatory conditions are multifactorial and range from chronic systemic inflammation (periodontitis) to the effects of risk factors such as diabetes, hyperlipidemia, smoking, and age which predispose to both cardiovascular diseases and oral disorders, such as periodontitis [1–5], caries [19–21], endodontic infections [22–26], or DRS [9, 12, 13]. This coincidence of risk factors is visible in the population of patients we have studied. Although the difference in occurrence of smoking or diabetes did not reach statistical significance, we can clearly see increased occurrence of these factors in DRS. This can in part explain the increased degree of endothelial dysfunction in DRS subjects. Measurement of baseline FMD prior to developing DRS in a long-term follow-up study would unquestionably strengthen the conclusions of this study. Alternatively, a future interventional study in which the effect of treatment of DRS on endothelial function could also help to address this issue in a more cause-effect manner. Importantly, as the population we studied was relatively young for denture carriers, no significant increase in intima-media thickness was detected yet. This is in agreement with numerous cardiovascular studies which show that endothelial dysfunction precedes the development of severe atherosclerosis [38].

The role of systemic inflammation, very well defined in periodontitis, is not known in DRS. The mechanisms through which DRS could affect endothelial dysfunction are unclear. In periodontitis, bacteria lead to the activation of the local immune response, leading to systemic inflammation. Similarly, immune stimulation of T cells and monocytes has been reported in response to fungal* C. albicans* antigens [39, 40]. However, in our study we did not find significantly increased levels of total CRP, which could suggest that local* Candida*-evoked oral mucosal inflammation is not causing significant activation of systemic inflammatory response. The CRP levels among edentulous were assessed by Ajwani et al. at Helsinki Aging Study involving over 600 patients older than 75 years old [41]. They identified mucosal lesions in the edentulous as an important factor associated with elevated CRP level among elderly individuals and observed that it was significantly more common among the edentulous with complete dentures. Importantly, patients having clinical signs of oral candidosis or denture stomatitis also showed elevated levels of CRP, and authors suggested that it may be the explanation of the elevated CRP levels seen in the edentulous. In our study, we have also seen a trend toward higher CRP values in DRS patients, but it did not reach statistical significance, probably because of small numbers of patients involved and the fact that we have not measured high sensitivity hsCRP which would better characterize cardiovascular risk [42]. In the light of our results, assessing other markers of the systemic inflammation becomes a very interesting aspect for further studies. Taking into account our results, at present our data do not support the hypothesis that systemic inflammation is involved. Rather, the effect of concomitant risk factors on DRS and vascular function is most likely.

De Oliveira et al. and Rodriguez-Archilla et al. have found that oral* Candida* infection may impact peripheral blood mononuclear cells state, measured by amount of cytokines produced in vitro in response to* Candida* antigens [39, 40]. Direct impact of* Candida* cells on the vascular system is unlikely, since systemic fungal infections are characterized, in contrast to the DRS, by extremely serious symptoms; moreover, fungal DNA has not been detected in atherosclerotic lesions [43].

However our results may point us to the potential role of vascular risk factors such as diabetes, smoking, and hypertriglyceridemia in mediating vascular dysfunction. Although no relationship was found between triglyceride levels and endothelial dysfunction in a simple correlation analysis, when multivariate linear regression was introduced including diabetes, smoking, and/or triglyceride levels, the difference in endothelial function was no longer significant (data not shown). One has to however bear in mind that statistical power of such analysis in the studied group was relatively modest.

While the finding that classical risk factors may be main mediators of endothelial dysfunction may not sound exceptionally interesting, it is very important to show that this is the case in DRS subjects, and therefore this group of patients should be carefully studied in larger epidemiological trials. This is particularly important in the light of ageing population.

Despite the lack of differences in the levels of total, HDL, and LDL cholesterol in the blood of healthy subjects and DRS, we observed significantly higher levels of triglycerides in patients with DRS. Our surprising finding that DRS is associated with selective increase in triglyceride levels is quite intriguing and could be related to the fact that denture wearing can change dietary habits. It could also suggest that increased triglyceride levels could be a risk factor of DRS, although our study was not powered to answer this question. There are no studies looking at lipid parameters in DRS, while conflicting data are available regarding periodontitis. Sandi et al. and Penumarthy et al. observed higher concentrations of total cholesterol and LDL cholesterol in the blood of patients with periodontal disease than in healthy group, but the differences in the levels of triglycerides and HDL cholesterol were shown only by Penumarthy et al., despite the smaller sample sizes [44, 45]. Simultaneously, Elter et al. did not demonstrate changes in the total cholesterol and HDL levels after treatment of periodontitis [37]. Altogether, these results can point to a potential relationship between oral infection and blood lipid profile, but conclusive evidence is still needed.

We did not observe the tendency towards elevated blood pressure in patients with DRS as compared to healthy subjects. This shows potential important difference in oral inflammatory conditions as periodontitis is potentially associated with elevated blood pressure, which was frequently observed [46]. The relationship between the use of dental prostheses and the prevalence of cardiovascular diseases, including elevated blood pressure, was discussed by Buhlin et al. [2]. They found positive association between dentures and all cardiovascular diseases but not for elevated blood pressure, myocardial infarction, or stroke. However, the group defined as “dentures” was very heterogeneous. The authors gathered together edentulous and partially edentulous denture wearers and edentulous without dentures. This creates potential bias of the presence of periodontitis in partially edentulous patients. Importantly, in our study virtually all subjects were completely edentulous (41 out of 44).

## 5. **Conclusions**

In conclusion, our study shows that patients with denture-related stomatitis are characterized by more pronounced systemic endothelial dysfunction than denture subjects without stomatitis. This difference in vascular function is likely linked with increased cardiovascular risk in DRS and indicates that such patients should be carefully monitored for cardiovascular disease. While our study identifies certain very interesting and potentially very important cardiovascular aspects of denture-related stomatitis, a larger study is warranted to finally confirm these observations. This may be very important for clinical practice considering ageing population.

## Figures and Tables

**Table 1:** Patient clinical characteristics.

	DRS group *n* = 20		Control group *n* = 24
Gender (M : F)	2 : 18	*P*> 0,05	6 : 18
Age [mean (SD)]	63,9 (6,6)	*P*> 0,05	65,9 (10,3)
BMI [median (*Q*1; *Q*2)]	28,5 (24,9; 33,6)	*P*> 0,05	27,8 (24,3; 29,3)
Smoking (%)	6 (30%)	*P*> 0,05	3 (12,5%)
Diabetes mellitus (%)	6 (30%)	*P*> 0,05	2 (8,3%)
Hypertension (%)	17 (85%)	*P*> 0,05	19 (79,2%)
Hyperlipidemia (%)	13 (65%)	*P*> 0,05	12 (50%)
Medications (%)			
ACE inhibitor	7 (35%)	*P*> 0,05	12 (50%)
Acetylsalicylic acid	3 (15%)	*P*> 0,05	4 (17%)
*β*-blocker	7 (35%)	*P*> 0,05	10 (42%)
Ca antagonist	3 (15%)	*P*> 0,05	8 (34%)
Diuretic	7 (35%)	*P*> 0,05	6 (25%)
Statin	6 (30%)	*P*> 0,05	5 (21%)
Insulin	2 (10%)	*P*> 0,05	2 (8,3%)
Oral antidiabetic agents	2 (10%)	*P*> 0,05	2 (8,3%)

ACE: angiotensin converting enzyme, BMI: body mass index, DM: diabetes mellitus, SD: standard deviation.

## Abbreviations

CRP: C-reactive protein
DM: Diabetes mellitus
DRS: Denture-related stomatitis
FMD: Flow-mediated dilatation
HDL: High density lipoprotein cholesterol
LDL: Low density lipoprotein cholesterol
NMD: Nitroglycerine-mediated dilatation
*Q*1 and* Q*3: 25th (*Q*1) and 75th (*Q*3) percentiles
SD: S: tandard deviation.
